# Preauricular Leiomyoma: A Rare Presentation of a Cutaneous Smooth Muscle Tumor

**DOI:** 10.7759/cureus.81695

**Published:** 2025-04-04

**Authors:** Lauren Velasquez, Daniel Hahn, Vijay Sookai, Bernard Pacella

**Affiliations:** 1 Medicine and Surgery, Touro College of Osteopathic Medicine, New York, USA; 2 Otolaryngology, Wyckoff Heights Medical Center, Brooklyn, USA

**Keywords:** cutaneous leiomyoma, facial tumor, leiomyoma cutis, preauricular mass, reed’s syndrome

## Abstract

Cutaneous leiomyoma, also known as leiomyoma cutis, is a rare presentation of an extra-uterine leiomyoma, often found on the extremities, trunk, and genitalia. Solitary lesions rarely occur on the face. This case presents a 63-year-old man with a one-year history of a growing mass in the preauricular region. On examination, a well-defined, painless mass was noted. Given the unusual yet visible location on the face, the decision was made to excise it surgically. After surgical resection, the biopsy specimen stained positively for smooth muscle actin (SMA), confirming a cutaneous leiomyoma. To the best of our knowledge, this is the eighth reported case of a preauricular cutaneous leiomyoma and stresses the importance of an accurate diagnosis for timely surgical treatment.

## Introduction

A leiomyoma is a benign smooth muscle tumor commonly found in the uterus, esophagus, and small bowel; however, less commonly, an extra-uterine leiomyoma can be found in cutaneous tissues. Cutaneous leiomyomas, or leiomyoma cutis, represent 5% of all leiomyomas; however, they represent 75% of all extra-uterine leiomyomas [1]. Pilomeiomyomas are often reported to form on the extremities, trunk, and genitals; however, more uncommonly, they can be found on the face [2]. 

Cutaneous leiomyoma is a benign, smooth muscle tumor that is subcategorized based on its origin: angioleiomyoma arises from the blood vessel’s tunica media, genital leiomyoma from the smooth muscles in the labia, scrotum, and nipple, and piloleiomyoma from the arrector pili muscle of the hair follicle [3]. Piloleiomyomas can occur sporadically or as part of Reed's Syndrome, an autosomal dominant genetic condition where piloleiomyomas develop and are associated with an increased risk for renal cell carcinoma [4]. Data regarding the incidence of cutaneous leiomyoma are limited; however, it is more common in adults than in children, and there is no incidence difference between biological sexes [5]. Based on limited cases, 9.3% of cutaneous leiomyomas are present on the face and neck, with most occurring on the trunk and limbs [1]. In this case report, a 63-year-old man presented with a piloleiomyoma in the preauricular area, the eighth reported case of this type of occurrence at the time of publication, and a rare and unusual location for this type of tumor.

## Case presentation

A 63-year-old male presented to the otolaryngologist with a right preauricular mass. He stated that he first noticed the mass one year ago, but it had grown recently. Due to its size and location, the patient met with the physician to have it removed. The patient denied pain on palpation of the mass, hearing loss, dizziness, or any other constitutional symptoms. Physical exam showed a mobile, soft 1 x 1 cm mass with smooth edges in the preauricular area. A radiological examination or needle aspiration biopsy was not performed, due to the unconcerned physical exam findings and lack of symptoms. No other masses on the body were reported. His past medical history included a breast imaging-reporting and data system (BI-RADS) category 2, benign gynecomastia mass in the right retroareolar region that was completely resected. The patient does not smoke. There was no family history of uterine fibroids, multiple cutaneous tumors, or renal malignancies. The patient returned for a one-week and one-month post-op visit with no reported recurrence of the mass; however, he has not returned for his scheduled three-month follow-up visit.

Operation report

The patient was placed supine and was administered IV sedation. A 2-cm incision was made vertically with a #15 blade in the preauricular area. Sharp and blunt dissection was carried down into the subcutaneous layer to identify the mass visually. The mass was then delineated horizontally by spreading it with Metzenbaum dissecting scissors. The mass was grasped gently with Allis forceps, which were used to facilitate dissection from its connective tissue attachments. A 1.2 x 0.8 x 0.4 cm mass was removed from the preauricular area. The patient tolerated the procedure well, with no complications.

Pathology report

**Figure 1:**
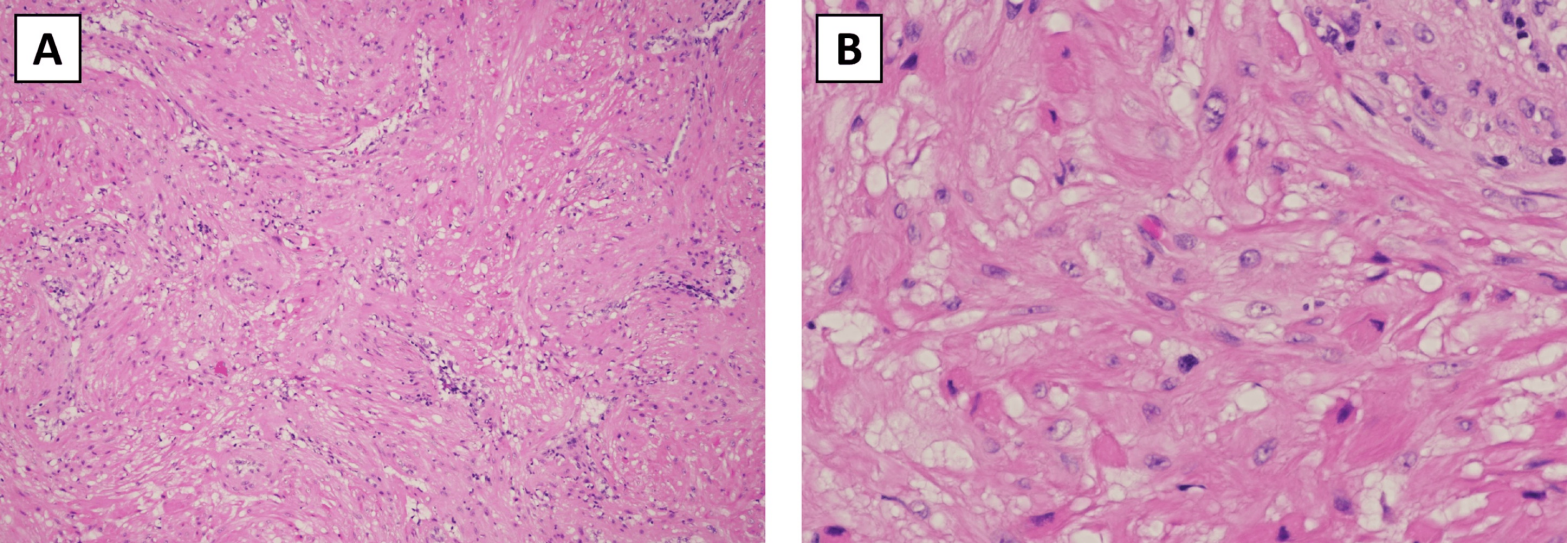
H&E stains of the leiomyoma The tumor showed (A) a whorled pattern and (B) spindle, "cigar-shaped" nuclei with blunt ends H&E: Hematoxylin and eosin

The pathologic report revealed findings consistent with cutaneous leiomyoma. The mass was described on gross inspection as a firm, smooth-surfaced, tan-white colored nodule that measured 1.2 x 0.8 x 0.4 cm in size. Bisectioning the mass revealed homogeneous tan-white surfaces. Hematoxylin and eosin (H&E) stain of the tumor showed a whorled pattern (Figure 1A) and spindle nuclei (cigar-shaped) with blunt ends (Figure 1B).

**Figure 2:**
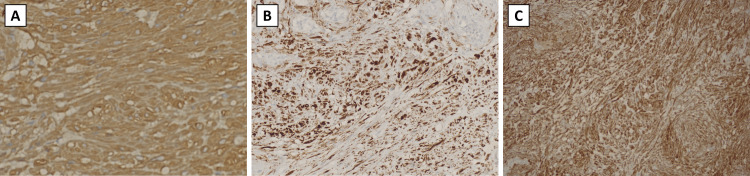
Positive immunohistochemistry stains for the leiomyoma (A) Smooth muscle actin stain; (B) Desmin stain; (C) Caldesmon stain

Immunohistochemistry staining was positive for smooth muscle actin (SMA), desmin, and caldesmon (Figures 2A-2C). The tumor stained negative for synaptophysin, CD34, factor XIIIa, β-catenin, and S100.

## Discussion

Leiomyomas are a common group of benign smooth muscle tumors typically found in premenopausal women, presenting as uterine fibroids. Leiomyomas are classically associated with the uterus and gastrointestinal tract, while cutaneous leiomyomas are often found on the skin of the extremities and the trunk. There are three subtypes of cutaneous leiomyomas: piloleiomyoma, genital leiomyoma, and angioleiomyoma. These can present as a solitary lesion or multiple lesions [4]. The piloleiomyoma is the most commonly presenting subtype and originates from the hair follicle’s arrector pili muscle. Based on the location in the present study, the solitary lesion tumor likely arose from an arrector pili muscle located in the preauricular region. Pilar leiomyomas typically present as non-specific, firm, reddish-brown nodules [6]. Nonspecific clinical presentation, especially in atypical locations such as the face and neck area, as seen in this patient, requires having broad differential diagnoses.

Differential diagnoses of preauricular mass lesions include the more commonly seen preauricular cysts or enlarged lymph nodes caused by immune stimulation. However, cancer, including the rare cutaneous leiomyomas, should be considered as well. A solitary cutaneous leiomyoma typically forms sporadically; however, multiple lesions of cutaneous leiomyomas may be noted in the setting of Reed’s Syndrome. Reed’s Syndrome is an autosomal dominant condition with an inherited mutation of the fumarate hydratase gene, which is commonly associated with multiple cutaneous leiomyomas and renal cell carcinoma [4]. Suspicion for this condition would involve a family history of tumors, especially uterine fibroids. In the present study, the patient had a past medical history of a unilateral mass in the right subareolar area. Leiomyoma of the breast is rare; however, if they present as breast lesions, they are often found in the subareolar region [7]. With our current patient, Reed’s Syndrome is unlikely due to the subareolar mass pathology report found to be of gynecomastia origin, a negative family history of tumors or uterine fibroids, and the patient reporting no renal or urinary symptoms.

Other differential diagnoses for preauricular lesions should include parotid gland masses, such as tumors, neoplasms, and cysts. Conditions involving the temporomandibular joint (TMJ) are also important to consider due to its anatomical proximity to the preauricular region. These conditions include ganglion cysts of the TMJ, as well as various bone lesions, such as osteomas, osteochondromas, and synovial chondromatosis. To perform an appropriate workup, a thorough history and physical exam should be conducted, along with CT/MRI imaging, which should be considered to rule out TMJ pathologies and neoplasms [8]. In our present case, due to the gradual progression over time, absence of constitutional symptoms, and benign findings on physical exam, the otolaryngologist opted for surgical excision.

Complete surgical resection is the gold standard treatment for solitary cutaneous leiomyomas [3]. Patients with multiple cutaneous leiomyomas have a high risk of recurrence [9]. The present study emphasizes the importance of testing smooth muscle tumor markers, such as SMA, in cutaneous and subcutaneous lesions to rule out cutaneous leiomyoma. A positive immunohistochemistry panel can alert diagnosticians to unusual symptom constellations, such as Reed’s Syndrome, and inform future treatment planning. This patient also presented with this lesion, primarily due to a cosmetic reason. This is an important consideration for lesions in highly visible areas, especially in the case of multiple cutaneous leiomyomas. Surgery may not be feasible for multiple lesions due to recurrence and cosmetic issues, with treatments including CO_2_ ablation [10]. Appropriate follow-up should include evaluation for recurrence and close dermatological observation for new lesions that may change treatment plans, as well as other considerations for genetic conditions.

## Conclusions

This case report highlights the rare occurrence of a cutaneous leiomyoma in the preauricular region, an atypical location for this type of tumor. It is imperative to be aware of the sporadic formation of solitary, benign cutaneous tumors on the face, because they are associated with an increased risk of hereditary conditions, such as Reed’s Syndrome. The findings in the case suggest that the physician should maintain a high index of suspicion towards rare tumors in unique locations, and emphasizes the need for thorough physical examinations and pathological evaluation to rule out malignancies.
